# Visualization of Actin Cytoskeleton in Cellular Protrusions in Medaka Embryos

**DOI:** 10.21769/BioProtoc.4710

**Published:** 2023-07-05

**Authors:** Toru Kawanishi, Ann Kathrin Heilig, Atsuko Shimada, Hiroyuki Takeda

**Affiliations:** Department of Biological Sciences, Graduate School of Science, University of Tokyo, Tokyo, Japan;

**Keywords:** Cellular protrusion, Actin, Confocal imaging, Microinjection, Medaka, Developmental biology

## Abstract

*C*ellular protrusions are fundamental structures for a wide variety of cellular behaviors, such as cell migration, cell–cell interaction, and signal reception. Visualization of cellular protrusions in living cells can be achieved by labeling of cytoskeletal actin with genetically encoded fluorescent probes. Here, we describe a detailed experimental procedure to visualize cellular protrusions in medaka embryos, which consists of the following steps: preparation of Actin-Chromobody-GFP and α-bungarotoxin mRNAs for actin labeling and immobilization of the embryo, respectively; microinjection of the mRNAs into embryos in a mosaic fashion to sparsely label individual cells; removal of the hard chorion, which hampers observation; and visualization of cellular protrusions in the embryo with a confocal microscope. Overall, our protocol provides a simple method to reveal cellular protrusions in vivo by confocal microscopy.

## Background

Cellular protrusions are fundamental structures that play pivotal roles in various aspects of developmental biology such as cell migration, cell-cell communication, and signal reception (Sanders et al., 2013; Roy et al., 2014; Sagar et al., 2015; Genuth et al., 2018; Mattes et al., 2018; Dalle Nogare et al., 2020; Bischoff et al., 2021; Heilig et al., 2022). These protrusions can be morphologically categorized into several types, including filamentous projections called filopodia, sheet-like structures called lamellipodia, and round-shaped blebs (Ridley, 2011). The distinct morphologies of cellular protrusions are manifested by the spatial distribution of cytoskeletal actin: in filopodia, for example, actin filaments elongate radially and change their length over time, while actin in lamellipodia constitutes a planar meshwork and exhibits treadmilling motions (Rottner et al., 2017). Thus, visualizing actin distribution is a good proxy to describe the morphology and dynamics of cellular protrusions. Actin distribution in living cells has been visualized with fluorescent probes, including actin-binding peptides/proteins such as LifeAct and Utrophin, and GFP-labeled actin itself (Melak et al., 2017). These actin probes are genetically encodable and therefore can be expressed in diverse types of cells and tissues via transgenesis or mRNA injection (Blaser et al., 2006; Riedl et al., 2010; Dalle Nogare et al., 2020). More recently, a new fluorescent actin probe called Actin-Chromobody was developed based on a single monomeric antibody (nanobody) against actin (Rocchetti et al., 2014; Panza et al., 2015). Use of this new probe could potentially minimize interference with the endogenous actin dynamics, which is sometimes observed for the traditional actin probes (Spracklen et al., 2014; Flores et al., 2019; Xu and Du, 2021).

Medaka (*Oryzias latipes*) is a freshwater fish with beneficial features for developmental biology, similar to zebrafish; daily spawning and external development of transparent embryos as well as a small genome size enable investigation of cellular events taking place during embryogenesis (Wittbrodt et al., 2002; Takeda and Shimada, 2010). Furthermore, there is a series of spontaneous and mutagenesis-mediated medaka mutants, some of which turned out to have unique mutations and phenotypes that have not been identified in zebrafish (Omran et al., 2008; Kawanishi et al., 2013; Porazinski et al., 2015), demonstrating that medaka is a model genetic organism complementary to zebrafish. Medaka thus offers an expanding opportunity for analyses of unprecedented cellular mechanisms underlying embryonic development.

Live imaging of cellular protrusions in an embryo requires its complete immobilization. In medaka, contractile movements of the yolk surface and the periderm occur between gastrulation and mid-somitogenesis stages (Yamamoto, 1975; Robertson, 1979; Fluck et al., 1983; Iwamatsu, 2004), which can be inhibited by administration of *n*-heptanol (Rembold and Wittbrodt, 2004). Moreover, as with zebrafish, trunk twitching caused by muscle contraction takes place later until the hatching stage (Iwamatsu, 2004). The twitching can also be blocked by tricaine treatment, although a long exposure to the drug leads to mild developmental defects (Swinburne et al., 2015). α-bungarotoxin, which blocks acetylcholine receptors, has been recently shown to efficiently inhibit muscle twitching in zebrafish and medaka embryos without perturbing embryonic development (Swinburne et al., 2015; Lischik et al., 2019).

Here, we describe a detailed protocol to visualize actin dynamics in cellular protrusions of medaka embryos step by step. We label actin filaments in some of the cells by introducing mRNA encoding Actin-Chromobody-TagGFP2 into a cell at the 4-cell stage. We also fully immobilize the medaka embryo while imaging at the subcellular level under a confocal microscope by injecting *α-bungarotoxin* mRNA as well. Since the expression of the fluorescent probe lasts up to 5–6 days, our method exploiting mRNA injection is a convenient way to visualize actin distribution during embryogenesis without the need to create transgenic lines. Our protocol should be applicable for a wide range of functional investigation of cell protrusions in living medaka embryos, as well as in zebrafish and other fish embryos that are amenable to microinjection and dechorionation.

## Materials and reagents

1. Medaka (*Oryzias latipes*) d-rR strain. Can be ordered from NBRP, Japan
  (https://shigen.nig.ac.jp/medaka/strainDetailAction.do?quickSearch=true&strainId=5666). Adult medaka should be kept at 26–28 °C in a room with a 14:10 h light/dark cycle to promote spawning
2. pMTB-*AC-TagGFP2* (Heilig et al., 2022). Available from the authors upon request
3. pMTB-*α-bungarotoxin* (Addgene Plasmid #69542) (Swinburne et al., 2015)
4. AvaI (New England Biolab, catalog number: R0152S). 10× CutSmart buffer is included in the product
5. EcoRV (New England Biolab, catalog number: R0195S). 10× CutSmart buffer is included in the product
6. 0.5% phenol red in DPBS (Sigma-Aldrich, catalog number: P0290)
7. Penicillin-streptomycin solution (Thermo Fisher Scientific, catalog number: 15140122)
8. Hatching enzyme (store at -80 °C). Can be ordered from NBRP, Japan (https://shigen.nig.ac.jp/medaka/strain/hatchingEnzyme.jsp). You can also make hatching enzyme by yourself [see pp. 254–255 in Kinoshita et al. (2009)]
9. Agarose (Funakoshi, catalog number: GA-001)
10. Agarose, low melting point (LMP) (Promega, catalog number: V2831)
11. Wizard SV gel and PCR clean-up system (Promega, catalog number: A9285)
12. mMESSAGE mMACHINE SP6 transcription kit (Thermo Fisher Scientific, catalog number: AM1340). 2× NTP/CAP, reaction buffer, and enzyme mix are included in the kit
13. RNeasy mini kit (Qiagen, catalog number: 74104)
14. Fish-scooping net with fine mesh
15. 9 cm plastic dish, Asnol (As One, catalog number: 1-8549-04)
16. Multidish, 4 wells, Nunc (Thermo Fisher Scientific, catalog number: 176740)
17. Glass bottom dish (Iwaki, catalog number: 3911-035)
18. Injection mold. You need to make the mold by yourself; to design it, see pp. 279 in Kinoshita et al. (2009). You can order a plastic mold using the design through the Shapeways website (https://www.shapeways.com/); choose “Smoothest Fine Detail Plastic” for the material
19. Glass capillary (Harvard Apparatus, catalog number: GC100F-10)
20. Microcapillary tip (Eppendorf, catalog number: 5242956003)
21. Forceps (Dumont, catalog number: No.5-INOX)
22. Pasteur pipette (Asahi Glass, catalog number: IK-PAS-9P)
23. Yamamoto’s Ringer’s solution (store at 4 °C) (see Recipes). Dilute 50 mL of 10× Yamamoto’s Ringer’s solution in 450 mL of water, autoclave it, and add 5 mL of penicillin-streptomycin solution
24. Hatching buffer (store at room temperature) (see Recipes). Dilute 100× hatching buffer in water

## Equipment

1. Heating dry bath incubator (Major Science, model: MD-MINI)
2. Heating chamber (e.g., Tokai Hit, model: INUC-KRi)
3. Microvolume spectrophotometer (Thermo Fisher Scientific, model: NanoDrop 2000c)
4. Capillary puller (Narishige, model: PC-10)
5. Microinjector (Eppendorf, model: FemtoJet 4i)
6. Manipulator (Narishige, model: M-152)
7. Magnet stand for the manipulator (Narishige, model: GJ-8)
8. Iron plate for the magnet stand (Narishige, model: IP)
9. Incubator (Mitsubishi Electric Engineering, model: CN-25C)
10. Stereomicroscope (Leica, model: M165 FC)
11. External light source for fluorescence excitation (Leica, model: EL6000)
12. Inverted confocal microscope (Zeiss, model: LSM 710)
13. 25× water immersion objective with a long working distance (Zeiss, catalog number: 420852-9871-000)
14. 40× water immersion objective with a long working distance (Zeiss, catalog number: 421867-9970-000)

## Software

1. Zen (Zeiss, https://www.zeiss.com/microscopy/en/products/software/zeiss-zen.html)
2. Fiji (https://imagej.net/software/fiji/) (Schindelin et al., 2012)

## Procedure

- **mRNA synthesis**
  mRNA is synthesized in vitro from a plasmid containing both a promoter sequence for SP6/T7/T3 RNA polymerase and an SV40-derived polyA signal. Here, we use the pMTB vector, which was originally designed for zebrafish transgenesis as well as in vitro mRNA synthesis (Wagner et al., 2018). pCS2+ is another vector that is commonly used for in vitro mRNA synthesis.
  1. Linearize the pMTB-*AC-TagGFP2* and pMTB-*α-bungarotoxin* plasmids with restriction enzymes. To do so, mix the following solutions and incubate at 37 °C for 2 h:
    Plasmid (pMTB-*AC-TagGFP2* or pMTB-*α-bungarotoxin*), 2 μg
    10× CutSmart buffer, 2 μL
    Restriction enzyme (EcoRV for pMTB-*AC-TagGFP2*, AvaI for pMTB-*α-bungarotoxin*), 1 μL
    Water up to 20 μL
  2. Run electrophoresis with 1 μL of the reaction solution to verify that the plasmids have been fully linearized.
  3. Purify the linearized templates with Wizard SV gel and PCR clean-up system kit as per the manufacturer’s instructions. Elute the DNA in 25 μL of water.
  4. Assess the concentration of the eluents with a NanoDrop spectrometer. The concentration should be approximately 100 ng/μL.
  5. Synthesize mRNA using the mMESSAGE mMACHINE SP6 transcription kit. After thawing the kit solutions at room temperature (except the RNA polymerase enzyme mix), mix the solutions listed below (from top to bottom) at room temperature and incubate at 37 °C for 2 h:
    Nuclease-free water up to 10 μL (in total)
    2× NTP/CAP, 5 μL
    10× reaction buffer, 2 μL
    Template, 0.5–1 μg
    Enzyme mix, 1 μL
    While handling RNA, be sure to avoid RNase contamination by wearing gloves.
  6. Digest the DNA templates. Add 1 μL of TURBO DNase (included in the kit) and incubate at 37 °C for 15 min.
  7. Purify the mRNA with RNeasy mini kit following the manufacturer’s instructions and elute the mRNA in 30 μL of nuclease-free water.
  8. Assess the concentration of the mRNA eluents with a NanoDrop spectrometer. The concentration should be 100–500 ng/μL. The A_280_/A_260_ ratio of purified mRNA is typically approximately 2.0. The quality of mRNA can be further validated by agarose gel electrophoresis, whereby a single band with little smear will appear unless mRNA is degraded or unsuccessfully synthesized. The solution can be stored at -80 °C for months.
- **Preparation of injection tools**
  1. Injection gel
    - Pour 20 mL of melted 1% agarose dissolved in the hatching buffer into a 9 cm plastic dish and apply the injection mold onto the agarose before it solidifies. Trapping the air bubbles under the mold should be prevented by slowly sliding the mold onto the agarose surface with an angle of 45° (Figure 1A and 1B).
    - Once the agarose is solidified, remove the mold and pour the hatching buffer on the gel to prevent drying out (Figure 1C). The agarose gel can be stored at 4 °C for a few months and repeatedly used.
  2. Injection needles
    - Pull the glass capillaries using a Narishige capillary puller. Adjust the No. 2 heater adjustment parameter to 57.5 and set the mode selector knob to the *step 1* mode. This configuration provides an ideal needle shape for microinjection into medaka embryos.
    - Store the needles in a container so that the fragile tips do not touch anything (Figure 1D).
    **Figure 1.**
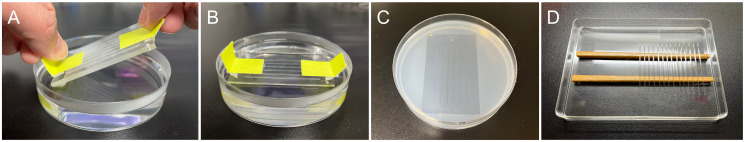
Injection tools for medaka embryos. (A, B) Injection mold sliding onto melted agarose. Note that the mold is gently put onto the agarose with an angle of 45° to exclude air bubbles between the mold and agarose (A). (C) Injection gel with 0.9 mm wide wells. (D) Injection needles stored in a container.
- **Microinjection into medaka eggs (Video 1)**
  **Video 1.**
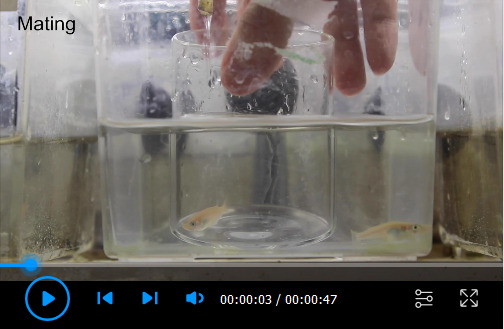
Microinjection into medaka eggs. After adult medaka pairs are crossed, collect fertilized eggs and remove attaching filaments on the chorion with a fish-scooping net to separate each other. Transfer separated eggs onto the injection gel and align them within the grooves of the gel. At the 4-cell stage, inject the mRNA solution into one of the cells.
  1. In the evening of the day before injection, pair adult medaka for mating in fish tanks. Separate females from males using transparent partitioners.
  2. In the morning of injection day (typically within 2 h after the room light turns on), remove the partitioner to enable the pairs to start mating. Mating will happen typically within 5–10 min.
  3. While waiting for the eggs (approximately 10–30 min), prepare the injection solution by mixing the following ingredients:
    *Actin-Chromobody-TagGFP2* mRNA, 1.5 μg
    *α-bungarotoxin* mRNA, 0.25 μg
    0.5% phenol red, 0.75 μL
    Nuclease-free water up to 10 μL
  4. Collect eggs from the female and transfer them into the hatching buffer.
  5. Medaka eggs tend to stick together because of long attaching filaments on the chorion. To separate eggs, transfer them into a fish-scooping net and gently roll the eggs with the net between your fingers until the egg clumps disintegrate and all eggs are separated (Figure 2A–2C). During the process, some short attaching filaments are removed as well, and tiny holes are created on the chorion; these holes will allow hatching enzyme to enter the chorion and digest it (Procedure D).
  6. Transfer the eggs onto the injection gel, which is covered with the hatching buffer, and gently align the eggs within the wells using forceps (Figure 2D).
  7. Load the prepared glass needle with ~3 μL of the injection solution using a Microloader tip and attach the needle to the injector.
  8. Initiate the injector and adjust the injection pressure (Pi) and compensation pressure (Pc) to appropriate levels. During the injection process, the needle should neither take up the embryo medium nor eject the injection solution too fast. Pressure values vary between individual needles, but we typically set Pi and Pc to ~500 and ~150 hPa, respectively.
  9. Break the tip of the needle by gently scratching the surface of a chorion, until injection solution slowly leaks out of the tip.
  10. When the embryos reach the 4-cell stage (Figure 2E), inject the solution into the cytoplasm of one cell of each embryo. This will lead to mosaic expression of Actin-Chromobody-TagGFP2, herewith highlighting actin dynamics in individual cells. The injection volume should be up to approximately half the size of the cell (corresponding to 2–3 nL).
    **Figure 2.**
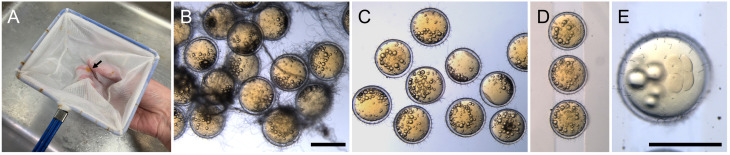
Separation of eggs using a fish-scooping net. (A) Gently roll the eggs (arrow) with a net to separate them. (B) Eggs with intact attachment filaments. (C) Eggs after separation using a net. (D) Eggs aligned on an injection gel. (E) A 4-cell embryo. Scale bars = 1 cm.
  11. Incubate the injected embryos in the hatching buffer at 23–32 °C.
- **Dechorionation**
  Dechorionation should be performed on the day of injection to maximize the chances of obtaining healthy embryos. Although dechorionation can be done later, digestion of the chorion is more likely to be incomplete, potentially leading to damaging the embryo with partially digested chorion pieces.
  1. Dilute the hatching enzyme 1:5–1:20 with 1/2× Yamamoto’s Ringer’s solution. The dilution ratio should be determined based on the enzymatic activity of the enzyme lot; when you use the lot for the first time, we recommend that you begin with a 1:20 dilution and examine how long it takes to digest most of the chorion (see step D3).
  2. Transfer embryos to a container with a small bottom area, e.g., a 4-well dish, and remove the culture medium (Figure 3A).
  3. Apply the diluted hatching enzyme onto the embryos and incubate them at 28 °C until the chorion is mostly digested (Figure 3B–3D). The incubation duration depends on the enzymatic activity of the lot, but usually takes up to 2–3 h. The enzyme will degrade the hard layer of the chorion, while a thin membranous layer will remain undigested (Figure 3D). It is recommended to check the digestion status in regular intervals (30–60 min). If the chorion is not digested within a few hours, try a higher concentration for the enzyme solution.
    **Figure 3.**
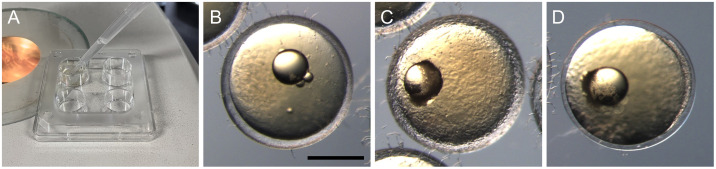
Dechorionation with hatching enzyme. (A) Apply diluted hatching enzyme solution onto embryos in a 4-well dish. (B-D) Temporal change of the chorion during hatching enzyme treatment. (B) Intact chorion has a thick hard layer. (C) Partially digested chorion shows a rough surface inside. (D) Digested chorion retains a thin membranous layer that can be torn with forceps. Scale bar = 500 μm.
  4. Carefully transfer the embryos to a glass dish filled with Yamamoto’s Ringer’s solution to rinse off the enzyme. Most of the embryos are still surrounded by the thin layer of the chorion, but some may be fully out of it. Transfer all embryos to a new glass dish filled with Yamamoto’s Ringer’s solution. Since physical contact with the bottom surface of a brand-new plastic dish could damage the embryo, keeping the embryos in a glass dish is preferred.
  5. Incubate the injected embryos at 23–32 °C until they reach the desired developmental stage. For stage 28, it takes approximately three days at 26 °C (Iwamatsu, 2004).
- **Mounting embryos in agarose for imaging**
  In this protocol, we focus on embryos from stage 28 onward.
  1. When the embryos reach the desired stage, screen for embryos exhibiting GFP fluorescence at the region of interest (ROI), such as somites or epidermis. For the screening, use a fluorescent stereomicroscope and transfer suitable embryos into a new dish filled with Yamamoto’s Ringer’s solution. Using forceps, gently remove the remaining chorion of the selected embryos.
  2. Dissolve 1% LMP agarose in hot Yamamoto’s Ringer’s solution and keep it at ~40 °C using a heating incubator.
  3. Pour a few drops of the LMP agarose solution to cover the entire glass part of a glass bottom dish and immediately transfer 2–3 embryos into the agarose. Mount the embryos before the agarose solidifies. Orient the embryos with forceps in a way that the ROI is in contact with the glass bottom (Figure 4). This allows the ROI to be within working distance of the confocal microscope. Once the agarose solidifies (within a minute), avoid moving the embryos. If the orientation of the embryos is not adequate, add Yamamoto’s Ringer’s solution to the dish, break the agarose with forceps to release the embryos, and repeat the mounting process.
    **Figure 4.**
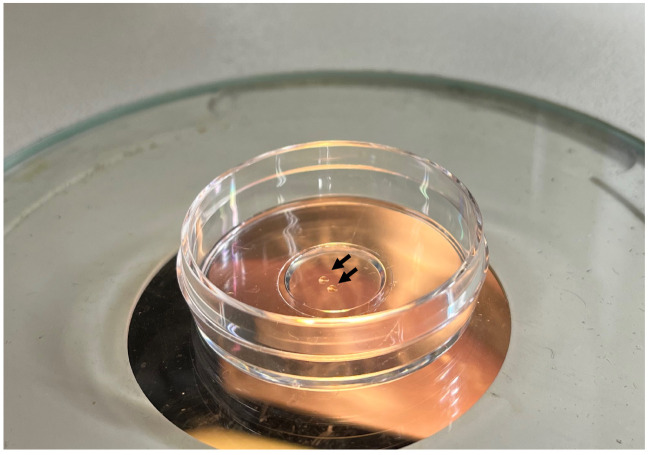
Mounting of embryos in low-melting-point agarose for imaging. Embryos are mounted adjacently to the glass surface of the dish (arrows) for subsequent imaging with an inverted confocal microscope.
- **Imaging cellular protrusions using an inverted confocal microscope**
  Using an objective with a long working distance is critical for imaging cells located inside the embryo.
  1. Start the confocal microscope and the microscope software Zen. Use the 25× water immersion objective and select the Locate tab on the software to allow observation through the eyepieces.
  2. Place the dish with the mounted embryos on the microscope stage and find the ROI by visual inspection through the eyepieces. Screen the embryos by their GFP fluorescence at the ROI.
  3. Once a good candidate is found, select the Acquisition tab on the software and start imaging the focal plane. Overall, tissue structures can be captured with this magnification.
  4. To obtain subcellular resolution, switch to the 40× water immersion objective and acquire images (Figure 5). Timelapse imaging can also be performed by activating the Time series checkbox on the software (Figure 6). If timelapse imaging takes a few hours or more, install a heating chamber on the stage to maintain the incubation temperature.
    **Figure 5.**
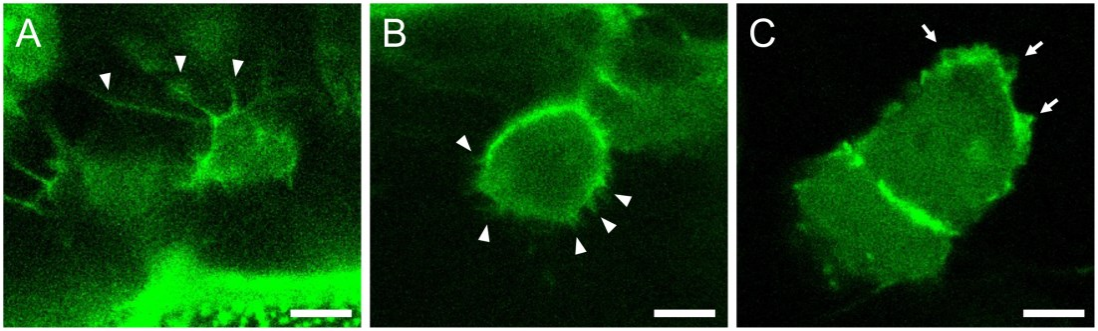
Representative images of cellular protrusions in different cell types. Actin-Chromobody-TagGFP2 (green) highlights cellular protrusions in a dorsal-most dermomyotomal cell (A), a dermomyotomal cell located more ventrally (B), and epidermal cells (C) of medaka embryos at stage 28. Arrowheads and arrows indicate filopodia and lamellipodia, respectively. Scale bars = 5 μm.
    **Figure 6.**
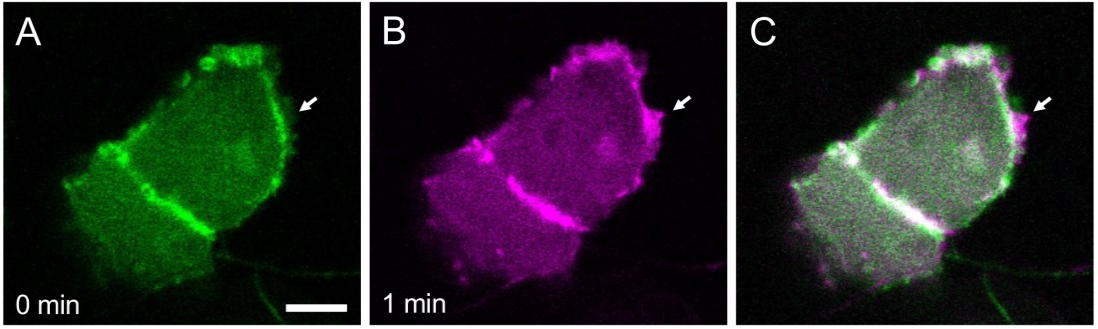
Temporal dynamics of cellular protrusions. Actin-Chromobody-TagGFP2 labels actin dynamics in epidermal cells of a medaka embryo at stage 28 (A) and one minute later (B). The merged image is shown in (C). Arrow indicates growth of a lamellipodium. Scale bar = 5 μm.

## Data analysis

- **Addition of a scale bar to the confocal image**
  1. Open the image in Fiji.
  2. Go to Analyze > Tools > Scale bar… and choose from displayed options. A scale bar with the defined length will appear on the image.

## Recipes

1. **10× Yamamoto’s Ringer’s solution (store at room temperature)**
  **Table:** 

Reagent	Final concentration	Amount
NaCl	1.3 M (7.5%)	75 g
KCl	27 mM (0.2%)	2.0 g
CaCl_2_·2H_2_O	18 mM (0.27%)	2.7 g
NaHCO_3_	24 mM (0.2%)	2 g
H_2_O	n/a	Up to 1,000 mL
Total	n/a	1,000 mL
2. **100× Hatching buffer (store at room temperature)**
  **Table:** 

Reagent	Final concentration	Amount
NaCl	1.7 M (10%)	100 g
KCl	40 mM (0.3%)	3 g
CaCl_2_·2H_2_O	27 mM (0.4%)	4 g
MgSO_4_·7H_2_O	65 mM (1.6%)	16 g
H_2_O	n/a	Up to 1,000 mL
Total	n/a	1,000 mL
